# Microbial and abiotic controls on mineral-associated organic matter in soil profiles along an ecosystem gradient

**DOI:** 10.1038/s41598-019-46501-4

**Published:** 2019-07-16

**Authors:** Robert Mikutta, Stephanie Turner, Axel Schippers, Norman Gentsch, Sandra Meyer-Stüve, Leo M. Condron, Duane A. Peltzer, Sarah J. Richardson, Andre Eger, Günter Hempel, Klaus Kaiser, Thimo Klotzbücher, Georg Guggenberger

**Affiliations:** 1 Soil Science and Soil Protection, Martin Luther University Halle-Wittenberg, Von-Seckendorff-Platz 3, 06120 Halle (Saale), Germany; 2 Leibniz Universität Hannover, Institut für Bodenkunde, Herrenhäuser Str. 2, 30419 Hannover, Germany; 3 Bundesanstalt für Geowissenschaften und Rohstoffe, Stilleweg 2, 30655 Hannover, Germany; 4 Agriculture and Life Sciences, Lincoln University, PO Box 85084, Lincoln, 7647 Christchurch New Zealand; 5 Landcare Research, PO Box 40, Lincoln, 7640 Canterbury New Zealand; 6 Institute of Physics, Martin Luther University Halle-Wittenberg, Betty-Heimann-Str. 7, 06120 Halle (Saale), Germany

**Keywords:** Carbon cycle, Biogeochemistry, Carbon cycle

## Abstract

Formation of mineral-organic associations is a key process in the global carbon cycle. Recent concepts propose litter quality-controlled microbial assimilation and direct sorption processes as main factors in transferring carbon from plant litter into mineral-organic associations. We explored the pathways of the formation of mineral-associated organic matter (MOM) in soil profiles along a 120-ky ecosystem gradient that developed under humid climate from the retreating Franz Josef Glacier in New Zealand. We determined the stocks of particulate and mineral-associated carbon, the isotope signature and microbial decomposability of organic matter, and plant and microbial biomarkers (lignin phenols, amino sugars and acids) in MOM. Results revealed that litter quality had little effect on the accumulation of mineral-associated carbon and that plant-derived carbon bypassed microbial assimilation at all soil depths. Seemingly, MOM forms by sorption of microbial as well as plant-derived compounds to minerals. The MOM in carbon-saturated topsoil was characterized by the steady exchange of older for recent carbon, while subsoil MOM arises from retention of organic matter transported with percolating water. Overall, MOM formation is not monocausal but involves various mechanisms and processes, with reactive minerals being effective filters capable of erasing chemical differences in organic matter inputs.

## Introduction

Up to 90% of the organic matter in soil is tightly bound to minerals, particularly in deeper soil layers^1^. Knowledge on processes involved in the formation of mineral-associated organic matter (MOM) has been identified as key in understanding soil carbon sequestration and climate change mitigation^2^. Two general pathways of MOM formation have been advocated, either the sorption of microbial assimilates^3^ or the sorption of plant-derived compounds^4,5^. The individual contribution of these pathways and the controlling factors are still not fully resolved.

**Figure 1:**
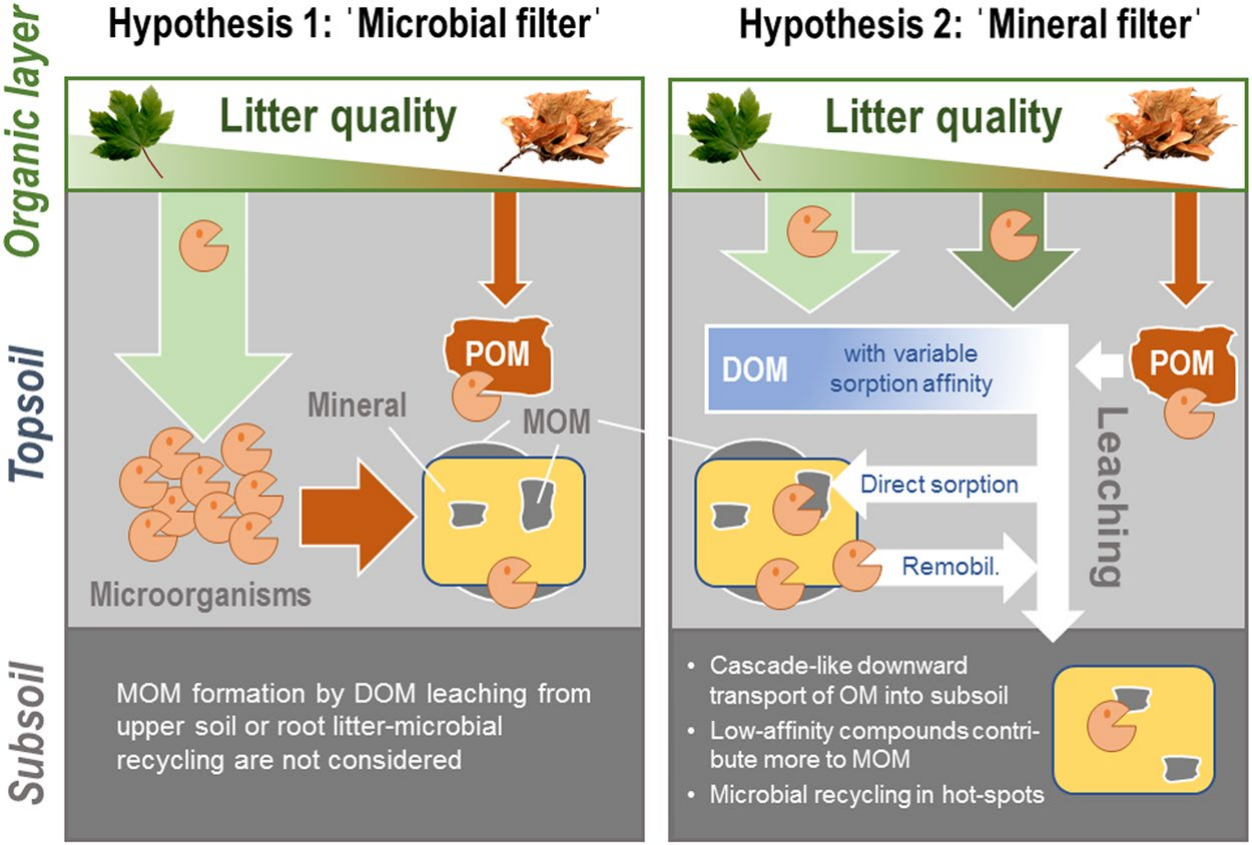
Opposing hypotheses on drivers of the formation of mineral-associated organic matter (MOM) in soils. The ‘microbial filter’ hypothesis (left) states that litter quality is the major factor of MOM formation with easily decomposable high-quality litter being preferentially assimilated by microorganisms due to higher substrate use efficiency^3^. Microbial assimilates then become sorbed to mineral surfaces^13^. Low-quality litter containing more structural lignin components is assumed to accumulate in mineral soils as residual particulate organic matter (POM)^13^. The ‘mineral filter’ hypothesis (right) presumes that microbial degradation of plant litter and POM results in a wide spectrum of dissolved organic matter (DOM) components that interact with mineral surfaces. At higher carbon occupation of mineral surfaces as in topsoils, strongly sorbing phenolic and proteinaceous compounds are preferentially retained while less strongly sorbing compounds such as polysaccharides are leached further down the profile. Large mineral surface occupation by organic matter also favors microbial colonization and activity. This may directly contribute to MOM formation in terms of released metabolites or necromass, but also cause re-mobilization of weakly bound MOM. The concept assumes that under conditions allowing for sufficient water transport through soil, the mineral phase developed during pedogenesis (abundance, type, and availability of reactive surfaces) and its carbon saturation level are the primary control to the formation of MOM and the share of microbial versus plant-derived components.

Cotrufo *et al*.^3^ proposed the ‘microbial efficiency-matrix stabilization’ framework to conceptualize the nexus from input and decomposition of plant litter to the formation of MOM. The framework assumes that the microbial community processes plant litter and then microbial assimilates become sorbed by minerals (‘microbial filter’ of MOM formation; Fig. 1). The efficiency of MOM formation is considered to depend on litter quality or, more precisely, on the carbon substrate use efficiency (i.e., the share of assimilated carbon that can be used for growth and enzyme production versus carbon being mineralized). The carbon substrate use efficiency is higher for labile compounds, such as carbohydrates and proteins, than for more stable litter components, such as lignin, and therefore relatively more microbial biomass is produced during the degradation of the labile compounds. The framework has received considerable attention^6–11^, although direct empirical evidence is restricted to short-term field and laboratory experiments covering a limited number of ecosystems^12–15^.

In a modeling approach, Castellano *et al*.^16^ showed that a pronounced effect of litter quality on MOM formation can only be expected if sufficient mineral sorption sites are available. The mutual effects of litter quality and mineral surface saturation on MOM formation might account for the uncertainty of recent soil organic matter models to predict carbon sequestration upon warming or altered litter inputs^17^. Sokol *et al*.^4^ proposed that litter-related microbial processing may be especially important in surface and rhizosphere soils, and in poorly drained soils. Conversely, direct sorption processes without preceding microbial assimilation of plant carbon may be more important in free-draining soils, supporting the vertical transport of dissolved organic matter. The downward migration of organic matter is supposed to involve repeated sorption and remobilization cycles^18^. Novel modeling approaches to predict ^14^C depth profiles, such as the COMISSION model^19^, account for such depth-dependent sorption processes. However, most experiments and modeling efforts on soil organic matter formation only focus on topsoils down to 0.3 m depth^13–15,17,20,21^. The influence of litter quality and related microbial assimilation on MOM formation throughout entire soil profiles is thus poorly understood, as is the contribution of direct sorption processes, mineral surface saturation, and the downward migration of organic matter (Fig. 1). This is highly relevant because most global soil organic carbon is stored in subsoil below 0.3 m (~60% of total soil carbon within 2 m)^22^.

Using a unique soil chronosequence along an ecosystem gradient spanning 120 ky at the Franz Josef Glacier in New Zealand, we investigated the pathways of MOM formation. This involved relating above- and belowground litter quality to amounts and composition of MOM along entire soil profiles. The study sites cover a complete sequence of ecosystem development^23^, with progressive stages characterized by sufficient nutrient (nitrogen and phosphorus) supply and increasing productivity until 12 ky, and retrogression stages, with exhausted nutrient reservoirs, especially of phosphorus^24,25^. The oceanic temperate climate (mean annual precipitation of ca. 6,500 mm at the four youngest sites and ca. 3,600 mm at the three oldest sites; mean annual temperature of ca. 10.8 °C)^25^ caused the development of podzolized soils with eluvial mineral topsoil horizons overlaying horizons being enriched in metal oxides and soil organic matter (Table 1).

**Table 1:** Site description and basic soil properties including diagnostic soil horizons.

Site age (ky)	Soil type	NDVI		Litter layer					Organic layers			Topsoils	Subsoils
		June 2017	Dec. 2018	C/N	VSC/N	Alkyl C/O-Alkyl C	Methoxyl C/O-Alkyl C	Horizon	C/N	VSC/N	Horizon	Horizon	Horizon
0.06	Haplic Regosols	0.8 (0.0)	0.8 (0.0)	26 (4)	2.2 (0.4)	0.3	0.1	Oi	15 (1)	0.4 (0.0)	OA	-	CA, C
0.5	Haplic Regosols	0.4 (0.1)	0.6 (0.0)	68 (7)	4.6 (1.2)	0.4	0.1	Oi	22 (4)	0.8 (0.5)	OA	A	CBg, Cg, Cr
1	Stagnic Regosols/Stagnic Cambisols	0.5 (0.1)	0.7 (0.0)	65 (27)	2.5 (0.6)	0.4	0.1	Oi	24 (3)	0.8 (0.0)	Oa	AE	Eg, Bg, Bs, BC
5	Stagnic Podzols	0.5 (0.0)	0.7 (0.0)	65 (15)	3.6 (1.2)	0.5	0.1	Oi	25 (2)	0.9 (0.1)	Oa	AE, AEg	Eg, Bg, Bs
12	Stagnic Podzols	0.4 (0.2)	0.7 (0.0)	85 (14)	3.3 (1.0)	0.4	0.1	Oi	35 (5)	1.2 (0.2)	Oa, Oe	AE	EA, EAg, Bh, Bs, B, Bw, C
60	Stagnic Podzols	0.3 (0.1)	0.7 (0.0)	105 (17)	5.5 (1.3)	0.4	0.1	Oi	35 (2)	1.6 (0.5)	Oe	AE, AEg	EAg, Bw, Bg, Bs, C
120	Stagnic Podzols	0.4 (0.1)	0.6 (0.0)	92 (12)	5.5 (0.7)	0.3	0.1	Oi	35 (6)	1.3 (0.3)	Oe	A, AE	Er, Eg, Bh, Bs, Bg, BC

The mean Normalized Density Vegetation Index (NDVI) at each site was calculated for June 2017 and December 2018 at a 10-m spatial resolution and the standard deviation is given for the number of pixels used per site (see Supplementary Information). Organic soil layers are represented by aboveground litter (Oi horizons) and organic horizons with intermediately (Oe) and highly decomposed (Oa) organic material. At the two youngest sites the organic material accumulated in transition OA horizons. The ratios of Alkyl C/O-Alkyl C and Methoxyl C/O-Alkyl C in litter samples were derived from CPMAS ^13^C-NMR spectroscopy (Supplementary Fig. S1). VSC refers to mass-based CuO-extractable lignin phenols (sum of vannilyl, syringyl, and coumaryl units). Values in parenthesis correspond to standard deviation of the mean of replicate samples.

## Results and Discussion

### Basic features of the ecosystem gradient

All sites were under temperate rainforest vegetation (see Supplementary Information) and Normalized Density Vegetation Index (NDVI) values obtained for June 2017 (winter) and December 2018 (summer) tended to decrease along the gradient, with differences among sites being more pronounced during winter (Table 1). The overall quality of surface plant litter (Oi horizons) changed markedly over 120 ky of ecosystem development, with high-quality litter (lower carbon-to-nitrogen and lignin-to-nitrogen ratios) dominating at the youngest site and low-quality litter (higher carbon-to-nitrogen and lignin-to-nitrogen ratios) at the older sites (Table 1). Solid-state ^13^C-NMR spectroscopy revealed only minor differences in carbon species among litter types (Table 1; Supplementary Fig. S1), making the nitrogen and phosphorus content the major distinguishing feature of litter chemistry along the gradient^26^. Soil mineralogy changed along the gradient, with greater clay stocks down to 1 m depth at the 60 and 120 ky site^27^. At younger sites (<1 ky), a greater portion of iron and aluminum resided in metal‒humus complexes, whereas older soils were enriched in poorly crystalline iron and aluminum oxide phases (Supplementary Table S1). Across all sites, the organic carbon stocks down to 1 m depth were positively correlated to oxalate-extractable aluminum, representing poorly crystalline aluminum phases and aluminum‒organic complexes (r = 0.83)^27^. In line with this, the microbial decomposability of soil organic matter was negatively related to the abundance of poorly crystalline iron and aluminum oxide phases as well as iron- and aluminum‒organic matter complexes (Supplementary Fig. S2 and Table S2).

**Figure 2:**
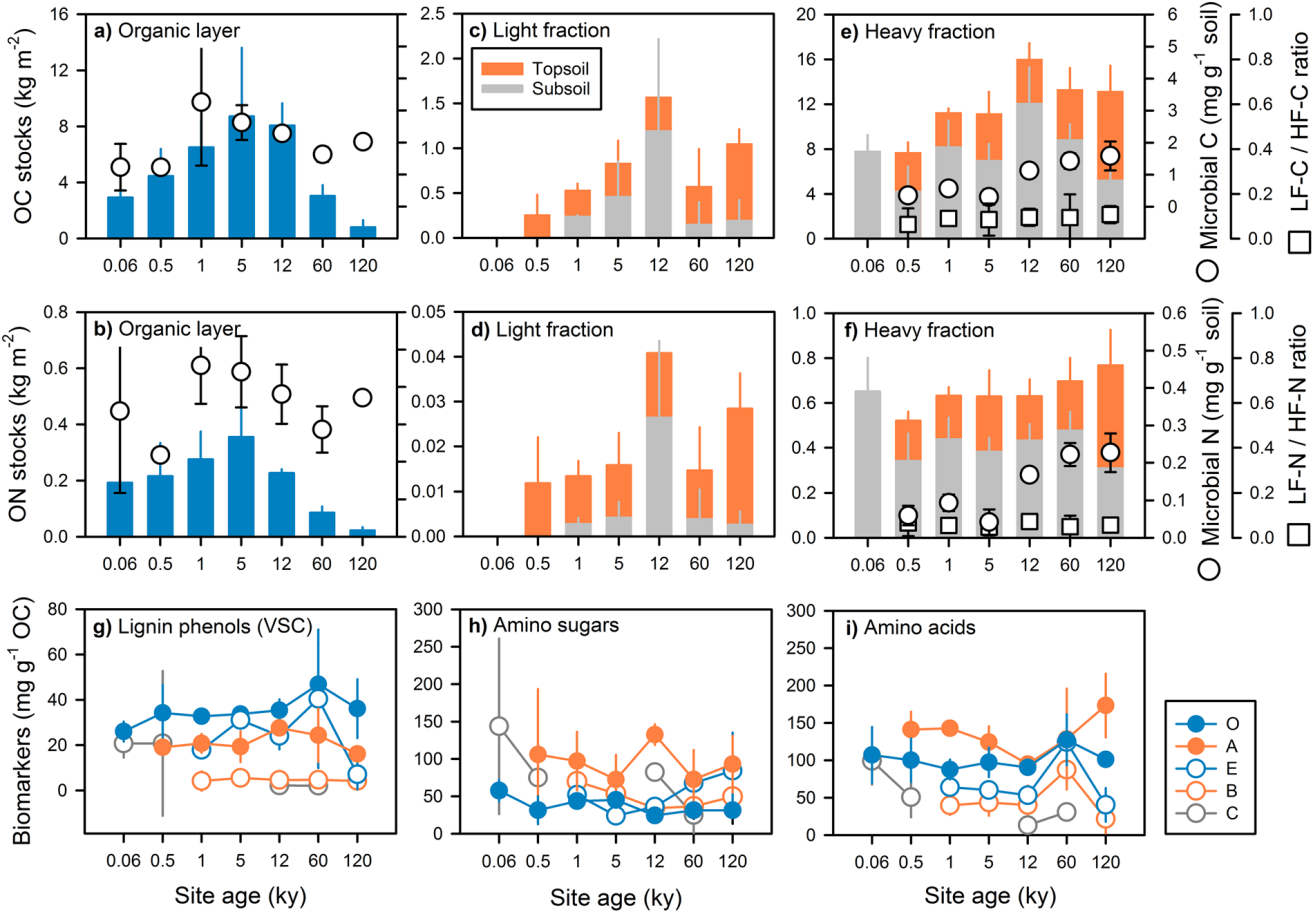
(**a** and **b**) Organic carbon (OC) and nitrogen (ON) stocks in organic layers (O horizons), (**c** and **d**) the light fraction (LF; i.e. particulate organic matter), and (**e** and **f**) the heavy fraction (HF; i.e., mineral-organic associations) of mineral topsoil (A horizons) and subsoil horizons (E, B, and C). Open circles in (**a**,**b**,**e** and **f**) represent microbial carbon and nitrogen in organic layers and topsoils whereas open squares in (**e** and **f**) represent the ratio of light fraction carbon to heavy fraction carbon in topsoils. Error bars represent standard deviation of replicated soil profiles (stocks) or soil horizons (microbial C and N, and light fraction carbon-to-heavy fraction carbon ratio). Error bars in case of the light fraction carbon-to-heavy fraction carbon ratio were calculated according to the rules of error propagation. Carbon-normalized concentrations of (**g**) lignin-derived VSC phenols, (**h**) amino sugars, and (**i**) amino acids in organic layers and heavy fractions of diagnostic soil horizons. Whiskers correspond to the standard deviation of the mean derived from replicated soil horizons. Note, there was no A horizon at the youngest site but a transition OA horizon, which was categorized as O horizon.

Stocks of organic carbon and nitrogen in organic soil layers increased towards the 5 ky site, and then, declined (Fig. 2a,b). Most of the organic carbon and organic nitrogen in mineral soil resided in the heavy fraction (>1.6 g cm^−3^), i.e., was associated with minerals (88 ± 11% for organic carbon; 93 ± 9% for organic nitrogen; mean ± SD; *N* = 80). The light fraction carbon, representing mainly fine root debris, accounted on average for 17 ± 8% of total carbon in topsoils and 11 ± 10% in subsoils; and it was virtually absent in half of the subsoil horizons.

**Figure 3:**
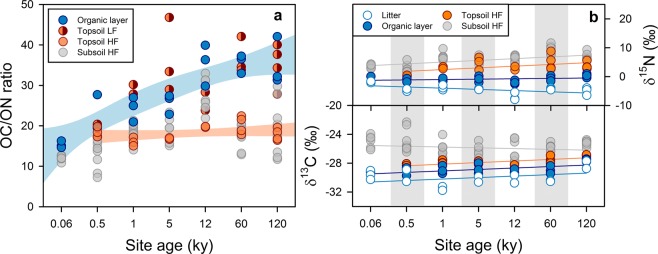
(**a**) Ratio of organic carbon-to-organic nitrogen (OC/ON) in organic layers (Oe, Oa, OA horizons) underneath aboveground litter, light fraction (LF) material of mineral topsoils as well as heavy fraction (HF) material of topsoils and subsoils along the Franz Josef chronosequence where aboveground litter quality decreases with site age. The blue and orange shaded areas indicate 95% confidence intervals of regressions of site age versus the OC/ON ratios of organic layers and HFs of topsoils. (b) Carbon and nitrogen isotopic composition of replicated aboveground litter (Oi horizons) and organic layers (Oe, Oa, OA horizons) as well as HF samples along the soil chronosequence. Individual data points denote replicated soil horizons. Linear regressions lines were included to depict major trends.

The organic carbon-to-organic nitrogen ratio of organic layers (Oe and Oa) increased from 15 ± 1 at the youngest to 35 ± 6 at the oldest site (Fig. 3a), which corresponded to the decrease in surface litter quality. The organic carbon-to-organic nitrogen ratio of the respective light fractions in topsoil increased also with site age (Fig. 3a), which in turn resulted in a reduced microbial decomposability (Supplementary Fig. S3). These results are consistent with general litter decomposition trends at other chronosequences^28^. The declining resource availability in the organic layers along the Franz Josef chronosequence is also reflected by changing invertebrate diversity and trophic organization^29^, microbial abundance, and extracellular enzyme activities^27^. Nonetheless, the organic layers as well as the mineral topsoil horizons along the sequence comprised comparable ratios of *Fungi* to *Bacteria* as based on SSU rRNA gene copy numbers, whereas *Archaea* were enriched in the oligotrophic subsoils, especially at older sites^30^.

### Lacking effect of litter quality on the formation of mineral-organic associations in topsoils

Topsoil horizons directly underneath the organic layer comprised invariable clay contents (12 ± 4%; *N* = 7), suggesting that already at the youngest sites the topsoil (including the OA horizon at the youngest site) offers a similar capacity for organic matter accrual as the topsoil of older sites. Given that transfer of carbon to MOM should be controlled by litter quality, topsoil at younger sites, with higher litter quality and phosphorus availability, should feature more mineral-associated carbon than those of older sites. However, the increasing carbon-to-nitrogen ratios of above- and belowground litter were not related to mineral-associated carbon (Fig. 2e; Supplementary Information ANOVA S1), and were also not related to average canopy height, number of woody species^25^, and tree biomass^28^. In addition, topsoil from all sites (except 12 ky) contained a similar ratio of mineral-associated carbon to clay stocks (i.e., 0.5 ± 0.1; *N* = 6). This also indicates there was no reduced accumulation of mineral-associated carbon at older sites with lower litter quality (Fig. 2e).

Particulate organic material in topsoil of the younger sites exhibits a two-fold higher potential microbial decomposability than that from the oldest site (Supplementary Fig. S3). Faster microbial assimilation of such high-quality litter should foster MOM formation at the younger sites. However, the ratio of carbon (and nitrogen) in light versus heavy fractions in topsoil horizons was constant along the entire ecosystem gradient (Fig. 2e,f). This suggests that the ratio of particulate soil organic matter to MOM in topsoil was constant over time. Consequently, there was no impact of changing litter quality and soil nutrient status on the formation of MOM in topsoil horizons. Even under the potentially higher biomass input at the intermediate sites (estimated from basal tree area^29^) due to sufficient nitrogen and phosphorus supply, there was no consistent trend in the stocks of topsoil MOM (Fig. 2e). Likewise, the heavy fraction carbon should have been least at the oldest site, featuring low NDVI and basal tree area, lowest leaf and litter phosphorus contents^21^, poorest litter quality, and thus, least litter decomposability. But this was not observed.

There are several explanations for the lacking litter quality effect along the gradient: First, in forest ecosystems, the dissolved organic matter transported from organic layers into topsoil does not only originate from the litter layer but mainly from the underlying, more decomposed O horizons, which contribute organic compounds strongly sorbing to minerals, such as oxidized lignin fragments^31^. Second, a substantial part of the labile carbon mobilized from aboveground litter can already be mineralized in the soil solution before reaction with minerals^32^. Third, the mineral surfaces in topsoil might be covered with organic matter, preventing additional uptake of carbon ― despite more carbon being delivered by the decomposition of labile litter^16^. Instead, large mineral surface coverages would favor the retention of strongly sorbing compounds^33^.

### Composition of mineral-associated organic matter along the ecosystem gradient

Within the ‘microbial efficiency-matrix stabilization’ framework, a microbial filter connects decomposing litter and mineral surfaces, resulting in largely microbial MOM (Fig. 1). Plant and microbial biomarkers in MOM can be used to distinguish carbon been transferred to the minerals with or without preceding microbial assimilation. Microbial cell wall-derived amino sugars and protein-derived amino acids comprised a significant part of MOM in topsoil, suggesting the microbial re-synthesis of organic substrates (Fig. 2h,i). Yet, the accumulation of these compounds in MOM was not consistent with the gradient in litter quality with increasing site age. Plant-derived lignin phenols were present in all topsoil and subsoil MOM along the chronosequence (Fig. 2g), indicating that MOM does not exclusively form by uptake of microbial compounds but also by plant-derived materials bypassing microbial assimilation (Fig. 1). Since alkaline CuO oxidation extracts not all lignin sorbed to soil minerals^34^, the contribution of plant-derived compounds is likely underestimated.

Contents of microbial biomass carbon and nitrogen increased in topsoil along the gradient despite of declining litter quality (Fig. 2e,f), without apparent effects on mineral-associated amino sugars and acids (Fig. 2h,i). The declining above- and belowground litter quality with site age was not reflected in declining amounts of microbial amino sugars and amino acids or in increased proportions of plant-derived lignin phenols in topsoils (Fig. 2g to i). These missing trends, however, are consistent with the invariable topsoil clay contents and organic carbon-to-organic nitrogen ratios of topsoil MOM (Fig. 3a). If a general microbial filter was causing preferential loss of carbon and enrichment of nitrogen during soil organic matter transformation, MOM would become even more nitrogen enriched with increasing soil depth^1^. Surprisingly, the organic carbon-to-organic nitrogen ratio of subsoil MOM, especially at the >5 ky sites, was frequently greater than those in the corresponding topsoil. These ratios align more closely with those of organic layers and particulate organic matter (Fig. 3a) than subsoil microbial matter (average carbon-to-nitrogen ratio of 8 ± 8^27^). This finding points to sources of subsoil MOM other than microbial ones.

### Indications for mineral surface saturation and selective organic matter retention

As suggested recently^4^, microbial recycling and direct sorption likely reflect a continuum, with the relative contribution depending on microbial activity in a given soil volume, the predominant hydrological regime, and the availability of mineral sorption sites. The oceanic temperate climate along the Franz Josef chronosequence facilitates water leaching, and thus, vertical transport of dissolved organic matter within the soil profile. It is well established that certain organic compounds bind directly and more strongly to minerals than others^35^ and even displace them from binding sites^36^. Several studies showed that lignin-derived aromatic compounds sorb preferentially to soil minerals due to their high content of carboxyl groups (e.g., summarized in Klotzbücher *et al*.^37^). Sanderman *et al*.^32^ demonstrated that the composition of organic matter derived from chemically diverse litter was astonishingly similar after reaction with soil clay fractions (dominated by allophane, Fe oxides, smectite, illite) because of preferential uptake of lignin and exclusion of proteins. Such mechanistic experiments support the idea of minerals acting as filters and being capable of erasing the chemical differences in the source materials (‘mineral filter’ of MOM formation; Fig. 1). The observed decoupling of litter quality and biomarker composition of topsoil MOM along the Franz Josef chronosequence supports this view.

The larger carbon-normalized lignin contents in MOM of topsoil than subsoil (B and C) horizons support the hypothesis of preferential binding of strongly sorbing components in the topsoils (Fig. 2g). Strong accumulation in the topsoils was also observed for amino sugars and amino acids (Fig. 2h and i). These nitrogen-rich components are capable of direct binding to minerals either as single compounds or with dissolved organic matter^38,39^. Transfer to minerals via microbial assimilates or necromass is also possible. Preferential binding of strongly sorbing components in topsoil, as indicated by the biomarker analyses, is highly likely because most mineral surfaces along the gradient are substantially covered by organic matter, as deduced from X-ray photoelectron spectroscopy (Supplementary Table S3). The mineral surface area occupied by organic matter ranged from 38% to 53%, which is higher than for very young (<20 y) granitic soils developed on the forefields of the retreating Damma Glacier in Switzerland^40^ but corresponds to values reported for topsoils of productive natural grasslands developed on felsic, granitic river terraces over 0.1 to 3,000 ky^20^. However, not all mineral surfaces are available for carbon allocation as they can be chemically unsuitable or occluded within aggregates. Hence, reported topsoil carbon coverages may be high enough to trigger competition between sorbing organic matter components. The ^14^C signature of MOM in topsoils was modern along the ecosystem gradient, indicating short turnover times and continuous displacement of older mineral-bound organic matter by strongly sorbing recent compounds (percent modern carbon in topsoil: 100.0‒108.3%, and subsoil: 11.2‒95.4%; Supplementary Table S4 and Fig. S4). Data from the 460-ky sandy Australian Cooloola chronosequence support this explanation: In the upper soil, where the absence of reactive minerals (<1% clay) intensifies competition among organic matter components for reactive binding sites, the ^14^C signature was modern throughout^41^.

Preferential binding of strongly sorbing, ^13^C-depleted aromatic moieties in topsoil, results in ^13^C-enriched, less aromatic and less strongly sorbing dissolved organic matter^42^ that then becomes transported deeper into soil where it interacts with less organically coated mineral surfaces. In line with this, MOM revealed distinct compositional changes with soil depth from topsoil to subsoil, i.e., declining lignin, amino sugar and amino acid proportions (Fig. 2) as well as increasing δ^13^C and organic carbon-to-organic nitrogen ratios (Fig. 3a,b). Support for the direct sorption of plant-derived organic matter bypassing microbial assimilation also comes from the humid Hawaiian chronosequence on basalt (0.3 ky to 4.1 My), comprising evergreen rain forest vegetation. Plant-derived lignin phenols and hydroxybenzenecarboxylic acids were enriched in topsoil MOM due to preferential retention by poorly crystalline minerals and hydrolysable metals^43^. Soil leaching experiments also confirmed the selective loss of plant-derived carboxyl-rich aromatics from solution by direct sorption^44^ without the need for preceding microbial assimilation.

### Linking mineral-organic interactions to pedogenesis and vertical organic matter transport

While our data do not reveal a dependency of MOM formation on litter quality in topsoil, it reconciles the view that in topsoil both microbial re-synthesis and direct sorption contribute to the formation of MOM. Depth-dependent trends in amounts and composition of MOM along entire soil profiles, however, rather support the idea of soil organic matter migrating downwards via continuous sorption‒remobilization cycles^18^ (Fig. 1). These cycles involve the temporal immobilization of organic compounds by sorption, followed by microbial processing, and partial desorption of altered compounds into soil solution^36^. In this “cycling downwards” concept, the microbial processing of weakly mineral-bound organic matter might be initiated by microbial taxa colonizing organically coated minerals^36^. Incubation of topsoil MOM along the age gradient also demonstrated that a fraction of the mineral-bound organic matter can be re-mobilized and processed by microorganisms^45^. The high soil biological activity and annual precipitation of >3,600 mm along the chronosequence make dissolved organic matter transported with percolating water a major source for the formation of MOM, especially in subsoil. The stepwise vertical translocation of organic matter and the underlying exchange reactions at mineral surfaces as well as biotic mobilization mechanisms, however, warrant further investigations. As particulate organic material was oftentimes absent in deeper soil horizons, root litter decomposition with subsequent microbial assimilation can be considered unlikely as the dominant pathway of MOM formation in subsoil.

### Implications

Carbon storage in MOM is a decisive factor in the global soil carbon cycle. Litter decomposition with subsequent microbial assimilation as well as direct sorption processes have been proposed as major drivers of carbon allocation to mineral-organic associations, especially in topsoils. This study took a whole-soil-profile approach along a 120-ky soil chronosequence and the findings did not support a direct link between litter quality and MOM formation in topsoil and subsoil. When considering entire soil profiles, patterns in MOM chemical and isotopic composition are more congruent with the direct sorption concept. This pathway involves the selective retention of certain compound classes due to competition for limited sorption sites as well as the downward migration of dissolved organic components. Our observations underpin the important role of minerals and their carbon saturation level in determining the quantity and quality of soil organic matter. They also indicate that the formation of MOM is not monocausal but involves various mechanisms and processes, all likely responding differently to changes in environmental conditions. Research on MOM, therefore, should aim at a more holistic understanding that finally can give way to a better representation of processes and controlling factors in models of soil carbon turnover. Proper estimates of the contribution of microbial litter processing versus direct sorption by minerals to MOM formation (microbial versus mineral filtering) as related to pedogenesis and the hydrological regime would certainly be a major leap forward.

## Methods

### Site description and sampling

Triplicate soil profiles (0‒1 m depth) including organic layers per site were sampled in January 2012 and February 2014 at seven sites along the Franz Josef soil chronosequence (0.06, 0.5, 1, 5, 12, 60, and 120 ky) located on the west coast of the South Island of New Zealand. Woody plant diversity, tree height, and vegetation cover increase during ecosystem progression, and decline at the oldest sites during retrogression^25^. Soils formed in glacial and fluvio-glacial sediments from schist and greywacke deposited during repeated glacial advance and retreat over 120 ky. Chemical and physical soil properties are given in Table 1, the Supporting Information, and elsewhere^27,30^. Stock data were calculated based on horizon depth, stone content, and bulk density^27^.

### Density fractionation of soils and isotopes

The light and heavy fractions were separated for 80 mineral soils according to Golchin *et al*.^46^ using sodium polytungstate adjusted to a density of 1.6 g cm^‒3^. The mean recovery of organic carbon with the fractions was 90 ± 10% (mean value ± SD). Analysis of organic carbon and total nitrogen as well as isotopic composition in organic layers and heavy fractions were carried out in duplicates on ground samples using an Elementar IsoPrime 100 IRMS (IsoPrime Ltd., Cheadle Hulme, UK) coupled to an Elementar vario MICRO cube EA C/N analyzer (Elementar Analysensysteme GmbH, Hanau, Germany). The δ^13^C values are given relative to the Pee Dee-Belemnite standard while the δ^15^N values are presented relative to the atmospheric isotopic signature of nitrogen gas. The ^14^C content in heavy fractions was analyzed by accelerator mass spectrometry (HVEE, Amersfoort, The Netherlands) at the Max Planck Institute of Biogeochemistry (Jena, Germany). Data were analyzed according to Steinhof *et al*.^47^ and expressed as percent modern carbon (100% = 1950 AD)^48^.

### Microbial biomass

Carbon and nitrogen in microbial biomass was determined by the fumigation-extraction method as described in Turner *et al*.^27^.

### NMR spectroscopy

Solid-state CPMAS ^13^C-NMR spectra of homogenized litter samples were recorded on a BRUKER AVANCE III at 100.5 MHz with a proton spin-lock and decoupling frequency of 400 MHz. The proton nutation frequency was 80 kHz corresponding to a π/2 pulse duration of 3.12 µs. The cross-polarization time was 500 µs. Samples were spun with 10,000 Hz and between 2,000 and 4,000 scans were recorded. After baseline correction, the intensities of spectral regions were corrected for different CP efficiencies at different spectral regions^49^. For this purpose, for one sample, ^1^H *T*_1ρ_ as well as *T*_CH_ were estimated selectively for the regions.

### Organic matter composition in organic layer and heavy fractions

Lignin-derived phenols in organic layers and heavy fractions were analyzed after alkaline CuO oxidation^43^ whereas amino sugars and amino acids were determined after acid hydrolysis^50^. Lignin phenols were derivatized with a 1:1 mixture of pyridine and N,O-bis(trimethylsilyl) trifluoroacetamide and measured on an ion-trap GC-MS system (450-GC / 220-MS, Agilent Technologies, Inc., Santa Clara, CA, USA) equipped with a VF-5ms column (Agilent). The recovery of the internal ethylvanillin standard averaged 80%. Amino sugars (sum of glucosamine, mannosamine, galactosamine, and muramic acid) were analyzed by aldonitril acetate derivates using a GC equipped with a flame ionization detector (GC-2010, Shimadzu Corp., Tokyo, Japan) and a SPB-5 fused silica column. The average recovery of the myo-inositol internal standard was 76%. Amino acids were measured as N-pentafluoropropionyl-amino acid isopropyl esters and concentrations of individual 26 compounds (including enantiomers) were analyzed using an ion-trap GC-MS system (320-GC / 220-MS, Agilent) equipped with a Chirasil-L-Val column (Agilent). The mean recovery of the L-norvaline internal standard was 66%. More analytical details are given in the Supporting Information.

### Mineralization of bulk organic matter and light fractions from topsoils

Carbon and nitrogen respiration of bulk soils were determined in 90-day incubation experiments conducted under oxic and anoxic conditions at 25 °C by using bulk soil samples (<2 mm) of four chronosequence sites (0.5, 5, 60, and 120 ky). Concentrations of carbon dioxide were analyzed by GC-ECD (Shimadzu GC-2014, Kyoto, Japan, modified according to Loftfield *et al*.^51^. In another set, we incubated light fraction material isolated from topsoils over 125 days at 15 °C as described in Turner *et al*.^30^. More details are provided in the Supporting Information.

### Statistics

Differences between groups were tested using non-parametric Kruskall-Wallis ANOVA tests using Statistica Version 13 (Dell Inc., Tulsa OK, USA) because data lacked homogeneity of variances and were not normally distributed (even after log transformation). For topsoil horizons, we used site age as a group variable (reflecting also litter quality) and different variables representing MOM chemistry or stocks as dependent variables. Spearman rank correlation was used to test for correlations between variables. Differences were considered significant at *P* ≤ 0.05.

## Supplementary information

Supplementary information
