# Supplementary material for: Microbial and abiotic controls on mineral-associated organic matter in soil profiles along an ecosystem gradient
Source: Sci Rep. 2019 Jul 16;9:10294. doi: 10.1038/s41598-019-46501-4 (PMC6635608; doi:10.1038/s41598-019-46501-4)
Supplement: Supplementary file 1 — Supplementary information [file 41598_2019_46501_MOESM1_ESM.docx]

**Supporting Information**

**Microbial and abiotic controls on mineral-associated organic matter in soil profiles along an ecosystem gradient**

Robert Mikutta^1,2^, Stephanie Turner^3^, Axel Schippers^3^, Norman Gentsch^2^, Sandra Meyer-Stüve^2^, Leo M. Condron^4^, Duane A. Peltzer^5^, Sarah J. Richardson^5^, Andre Eger^5^, Günter Hempel^6^, Klaus Kaiser^1^, Thimo Klotzbücher^1^ & Georg Guggenberger^2^

^1^Soil Science and Soil Protection, Martin Luther University Halle-Wittenberg, Von-Seckendorff-Platz 3, 06120 Halle (Saale), Germany. ^2^Leibniz Universität Hannover, Institut für Bodenkunde, Herrenhäuser Str. 2, 30419 Hannover, Germany. ^3^Bundesanstalt für Geowissenschaften und Rohstoffe, Stilleweg 2, 30655 Hannover, Germany. ^4^Agriculture and Life Sciences, Lincoln University, PO Box 85084, Lincoln 7647, Christchurch, New Zealand. ^5^Landcare Research, PO Box 40, Lincoln 7640, Canterbury, New Zealand. ^6^Institute of Physics, Martin Luther University Halle-Wittenberg, Betty-Heimann-Str. 7, 06120 Halle (Saale), Germany. Correspondence and requests for materials should be addressed to R.M. (email: robert.mikutta@landw.uni-halle.de)

**Content**

***Material and Methods***

## Site description, sampling, and basic soil properties 4

## Density fractionation of soils 5

## Organic matter composition in organic layer and heavy fractions 5

## X-ray photoelectron spectroscopy 6

## Mineralization of bulk organic matter and light fractions from topsoils 6

***Tables and Figures***

**Table S1**: Calculated stocks of particle-size classes and mineralogical proxies in topsoils and subsoils along the Franz Josef chronosequence.

8

**Table S2:** Averaged mineralization data from 90-day incubation experiments using bulk soil materials under oxic and anoxic conditions.

9

**Table S3:** Average atomic% concentrations (at.%) of elements at mineral surfaces in heavy fractions of selected topsoils (A and AE horizons) and percentage occupation of mineral surfaces by organic matter as analyzed by X-ray photoelectron spectroscopy.

10

**Table S4:** Radiocarbon data of topsoil and subsoil heavy fractions from diagnostic horizons of selected sites.

10

**Fig. S1:** ^13^C-NMR spectroscopy data of surface litter collected at the different sites 11

**Fig. S2:** Plot of (**a**) oxalate-extractable Fe plus Al (Fe_o_+Al_o_) and (**b**) pyrophosphate-extractable Fe and Al (Fe_p_+Al_p_) versus the percentage of OC in topsoil and subsoil bulk soils mineralized in 90 days at 25 °C under oxic and anoxic conditions.

12

**Fig. S3:** Mineralization of light fraction material isolated from A horizons along the Franz Josef chronosequence via density fractionation and incubated over 125 days under oxic conditions at 15°C.

13

**Fig. S4:** (**a**) General relationship between the percent modern carbon (pMC) of heavy fractions and soil depth considering samples from the 0.5, 5, 12, and 120 ky site and (**b**) relationship of pMC values of topsoil heavy fractions and their contents of lignin-derived phenols (VSC), amino sugars (AS), and amino acids (AA).

14

***Statistics and References***

Kruskall-Wallis statistics (ANOVA S1 and S2) 15

Spearman rank correlations 20

References 21

## **Site description, sampling, and basic soil properties**

The Franz Josef soil chronosequence is located on the west coast of the South Island of New Zealand and characterized by a long-term decline in phosphorus availability^1,2^ and increasing N concentrations^3^ during ecosystem development. Soils formed in greywacke and mica schist debris deposited during repeated glacial advance and retreat over 120 ky. Micas and trioctahedral chlorites were the dominant phyllosilicates in the parent material. Vermiculite and several interstratifications with vermiculitic, smectitic, chloritic, and micaceous layers developed over time; kaolinite formed at older sites (>12 ky) and was most abundant at the oldest 120 ky site^4^. Soils are under temperate rainforest with a general dominance of evergreen angiosperms. Canopy dominant trees are the N fixer *Coriaria* *spec*. (one of the few deciduous species) at the 0.06 ky site, other evergreen angiosperms (*Metrosideros umbellata*, *Weinmannia racemosa*) at the 0.5 ky site, and evergreen conifers (mostly *Dacrydium cupressinum*) together with *Metrosideros* and *Weinmannia* at the older sites (5–120 ky)^5^. The NDVI data were calculated from satellite imagery of Sentinel 2A using bands 4 (red) and 8 (near-infrared (NIR)) captured on 6 June 2017 (austral winter) and 23 December 2018 (austral summer) with a spatial resolution (pixel size) of 10 m (European Space Agency^6^), and retrieved on 15 February 2019 from the Copernicus Open Access Hub (https://scihub.copernicus.eu/dhus/#/home). The NDVI was calculated with the formula NDVI = (NIR - red) / (NIR + red) using QGIS 3.2.3. After generating a circular buffer of 20 m radius around each site, the average of all pixels completely or partially contained in each buffer area yielded the representative site-specific NDVI value.

Per site we sampled triplicate soil profiles by horizon down to one meter depth. Horizons below the mineral A horizons were classified as ˈsubsoilsˈ. Basic chemical and physical soil properties are given in Table 1 and described in elsewhere^7,8^.

## **Density fractionation of soils**

The light and heavy fractions were separated for 80 mineral soils according to Golchin et al.^9^. Twenty-five gram of soil was dispersed in 125 ml sodium polytungstate (density 1.6 g cm^‒3^) solution in a 250-ml centrifuge bottle and sonicated under cooling with 60 J ml^‒1^ at 15 mm immersion depth. Then, the suspension was shaken for 10 minutes and left to settle for one hour. After centrifugation for 10 minutes at 4,000 *g*, the supernatant was filtered (Whatman® GF 6 glass fiber filter) and the light fraction was washed with doubly deionized (DD) water until the electrical conductivity was <50 µS cm^‒1^ and freeze dried. The heavy fraction was re-suspended in 200 ml DD water, shaken for 10 minutes, centrifuged for 30‒60 minutes at 4000 *g*, washed until the electrical conductivity was <50 μS cm^‒1^ and subsequently freeze dried. The mean recovery of organic carbon with the density fractions was 90 ± 10% (mean value ± standard deviation).

## **Organic matter composition in organic layers and heavy fractions**

Lignin-derived phenols in organic layers and heavy fractions were analyzed by alkaline oxidation^10^ and expressed by the sum parameter VSC which integrates over eight oxidation products [Vanillyl (V) units: vanillin + vanillic acid + acetovanillone; Syringyl units (S) = syringaldehyde + syringic acid + acetosyringone; Cinnamyl units (C) = ferulic acid + p-coumaric acid]. A gas chromatograph (GC) system (450-GC) equipped with a VF-5ms capillary column (30 m, 0.25 mm i.d., 0.25 µm film, Agilent Technologies, Inc., Santa Clara, CA, USA) coupled to an ion trap mass spectrometer (220-MS) was used for analysis. Amino sugars (sum of glucosamine, mannosamine, galactosamine, and muramic acid) in heavy fractions were determined after hydrolysis in 6 M HCl^11^. Hydrolysates were filtered and impurities precipitated by neutralization with 0.4 M KOH. Aldonitril acetate amino sugar derivates were analyzed using a GC equipped with a flame ionization detector (GC-2010, Shimadzu Corp., Tokyo, Japan) and a SPB-5 fused silica column (30 m, 0.25 inner diameter, 0.25-µm film; Supelco, Bellefonte, PA). Amino acids in organic layers and heavy fractions were determined after HCl hydrolysis in 6 M HCl^12^. Samples were purified by a preconditioned Dowex 50Wx8 resin (Dow Chemical, Midland, MI, USA) and metals were removed by rinsing with 0.1 M oxalic acid. After elution of amino acids by 2 M NH_4_OH, the dried residue was re-dissolved in HCl (pH <2), centrifuged at 5,000 *g* for 15 minutes, and transferred into 5-mL reactivials. Samples were freeze-dried and derivatized. The resulting N-pentafluoropropionyl-amino acid isopropyl esters were analyzed by an ion-trap GC-MS system (320-GC / 220-MS, Agilent) equipped with a Chirasil-L-Val column (25 m, 0.25 mm inner diameter, 0.12 µm film thickness; Agilent). The total amino acid concentration was calculated as the sum of glycine and following enantiomers (D/L-alanin, D/L-aspartic acid, D/L-glutamic acid, L-isoleucine, D/L-leucine, D/L-valine, D/L-lysine, L-methionine, L-ornithine, D/L-phenylalanine, D/L-proline, D/L-serine, L-threonine, L-tyrosine, and D/L-valine).

## **X-ray photoelectron spectroscopy**

X-ray photoelectron spectroscopy was applied to topsoil heavy fractions from four sites also used for incubation experiments (0.5, 5, 60, and 120 ky) and provided information on the average elemental composition and carbon loading of outermost particle surfaces within top ∼10 nm. Duplicate samples (<63 μm) were deposited onto adhesive copper-nickel tape and analyzed in 300 × 700 µm areas by a Kratos Axis Ultra DLD instrument (Kratos Analytical Ltd, Manchester, UK) in the energy range of 0–1200 eV by applying a pass energy of 160 eV, a step size of 1 eV, and three sweeps. Duplicate analyses were averaged and shown as mean value ± mean range.

## **Mineralization of bulk organic matter and light fractions from topsoils**

Carbon and nitrogen respiration of bulk soils were determined in 90-day incubation experiments conducted under oxic and anoxic conditions by using bulk soil samples (<2 mm) of four chronosequence sites (0.5, 5, 60, and 120 ky). To do so, 2.5 or 5 g dry soil for organic layers (Oe, Oa, OA horizons) or mineral horizons, respectively, were weighted into 125-ml serum vials and adjusted to 60% water-holding capacity with DD water. Bottles were closed with polyester wool to allow gas exchange, and placed into an incubator set to 25°C. Samples were pre-incubated to activate microbial activity for 8 days. The water content of the samples was checked every few days and, if necessary, re-adjusted by adding DD water. Anaerobic respiration was determined in closed bottles under pure helium (purity 5.0) atmosphere. For oxic gas sampling, bottles were closed and flushed with synthetic air either 8 hours (O horizons) or 25 hours (mineral horizons) before sampling. For anoxic gas sampling, the bottles were flushed with pure helium 160 to 200 hours before sampling. Headspace samples were taken with a 25-ml syringe and transferred into pre-evacuated 20-ml vials. Concentrations of carbon dioxide were analyzed by GC-ECD (Shimadzu GC-2014, Kyoto, Japan; modified according to Loftfield et al. (1997)^13^. The mineralization data were fitted to a double exponential model assuming a fast and slowly cycling carbon fraction^14^. Cumulative mineralization was calculated as the sum of all daily gas emissions, while days between the sampling dates were interpolated linearly. After 90 days, mineral nitrogen (N_min_) was measured by extraction with 1 M KCl and the net nitrogen mineralization was calculated as the difference in N_min_ before and after incubation.

Light fraction material isolated from topsoils by density fractionation was separately incubated for 125 days at 15 °C as described in Turner et al. (2017)^8^. Briefly, 1 g of light fraction material was mixed with 20 g of quartz powder (<125 mm, Carl Roth GmbH C Co. KG, Karlsruhe, Germany) in 125-mL serum vials and the water content was adjusted to 60% water holding capacity. The light fraction was inoculated with 3-ml soil slurry obtained by extracting topsoil material of a given site with a sterile Hoagland solution (without phosphorus or nitrogen source) at a soil weight-to-volume ratio of 1:10. Carbon dioxide emissions and data treatment was performed as described above.

**Table S1**: Calculated stocks of particle size classes and mineralogical proxies in topsoils and subsoils along the Franz Josef chronosequence. Fe_d_ = content of dithionite-extractable Fe (total pedogenic Fe), Fe_o_ = oxalate-extractable Fe (Fe in poorly crystalline Fe oxides and Fe-organic complexes; Fe_p_ = pyrophosphate-extractable Fe = Fe in Fe-organic complexes; Al_o_ = oxalate-extractable Al (Al in poorly crystalline Al phases and Al-organic complexes; Al_p_ = Al in Al-organic complexes). More details about the selective extraction methods are given in Turner et al.^7^; SD = standard deviation of the mean.

| Site age | Clay |  | Silt |  | Sand |  | Fe_d_ |  | Fe_o_ |  | Fe_p_ |  | Al_o_ |  | Al_p_ |  |
| --- | --- | --- | --- | --- | --- | --- | --- | --- | --- | --- | --- | --- | --- | --- | --- | --- |
| ky | kg m^‒^² | SD | kg m^‒^² | SD | kg m^‒^² | SD | kg m^‒^² | SD | kg m^‒^² | SD | kg m^‒^² | SD | kg m^‒^² | SD | kg m^‒^² | SD |
| Topsoil | | | | | | | | | | | | | | | | |
| 0.06 | ‒ |  | ‒ |  | ‒ |  | ‒ |  | ‒ |  | ‒ |  | ‒ |  | ‒ |  |
| 0.5 | 6.21 | 2.30 | 20.72 | 9.32 | 12.80 | 11.27 | 0.13 | 0.05 | 0.16 | 0.05 | 0.17 | 0.08 | 0.09 | 0.03 | 0.14 | 0.07 |
| 1 | 5.99 | 0.63 | 28.31 | 1.10 | 14.07 | 3.12 | 0.07 | 0.02 | 0.07 | 0.03 | 0.11 | 0.05 | 0.06 | 0.00 | 0.08 | 0.02 |
| 5 | 11.89 | 12.68 | 50.86 | 60.13 | 27.86 | 37.22 | 0.16 | 0.22 | 0.12 | 0.18 | 0.07 | 0.07 | 0.10 | 0.18 | 0.05 | 0.08 |
| 12 | 2.51 | 1.28 | 19.88 | 11.72 | 12.26 | 5.43 | 0.02 | 0.01 | 0.02 | 0.01 | 0.01 | 0.01 | 0.04 | 0.02 | 0.03 | 0.01 |
| 60 | 13.78 | 5.92 | 70.66 | 21.83 | 20.63 | 5.01 | 0.23 | 0.29 | 0.18 | 0.24 | 0.06 | 0.07 | 0.17 | 0.10 | 0.10 | 0.06 |
| 120 | 14.01 | 3.06 | 119.97 | 20.25 | 20.16 | 3.30 | 0.04 | 0.01 | 0.03 | 0.01 | 0.03 | 0.01 | 0.05 | 0.02 | 0.05 | 0.03 |
| Topsoil and Subsoil | | | | | | | | | | | | | | | | |
| 0.06 | 63.23 | 31.08 | 336.57 | 78.31 | 798.76 | 47.69 | 4.19 | 0.78 | 2.51 | 0.64 | 1.47 | 0.69 | 0.61 | 0.15 | 0.49 | 0.20 |
| 0.5 | 81.59 | 16.64 | 731.79 | 104.77 | 770.01 | 105.44 | 1.78 | 0.31 | 1.61 | 0.30 | 0.58 | 0.38 | 0.64 | 0.25 | 0.87 | 1.07 |
| 1 | 49.49 | 14.96 | 369.57 | 65.92 | 499.38 | 211.75 | 4.88 | 1.41 | 4.21 | 1.65 | 1.71 | 1.44 | 2.38 | 1.04 | 2.85 | 1.59 |
| 5 | 78.88 | 12.77 | 481.30 | 44.12 | 382.01 | 131.97 | 5.62 | 1.05 | 4.76 | 1.12 | 0.76 | 0.09 | 2.03 | 0.23 | 1.05 | 0.07 |
| 12 | 46.48 | 31.50 | 213.09 | 111.23 | 344.04 | 204.80 | 2.99 | 1.19 | 2.00 | 0.47 | 0.31 | 0.10 | 3.65 | 2.11 | 1.25 | 0.60 |
| 60 | 114.04 | 26.25 | 478.94 | 43.32 | 271.72 | 77.13 | 4.93 | 2.53 | 3.83 | 1.65 | 0.65 | 0.07 | 4.34 | 2.62 | 1.85 | 0.80 |
| 120 | 179.25 | 64.53 | 841.32 | 289.79 | 226.85 | 32.12 | 8.26 | 2.12 | 1.42 | 0.59 | 0.70 | 0.45 | 4.18 | 2.14 | 1.85 | 0.90 |

**Table S2:** Average mineralization data from 90-day incubation experiments using bulk soil materials under oxic and anoxic conditions. For details of the experimental settings please refer to the experimental section; Abbreviation: Hor.= horizon.

| Site age | Hor. | Net-N-Min. | Net-N- Min. | CO_2_-C | CO_2_-C | CO_2_-C anox. | CO_2_-C anox. |
| --- | --- | --- | --- | --- | --- | --- | --- |
| (ky) |  | ox. (mg g^‒1^) | anox. (mg g^‒1^) | (mg C g^‒1^ soil) | (mg C g^‒1^ OC) | (mg C g^‒1^ soil) | (mg C g^‒1^ OC) |
| 0.5 | OA | 0.362 | 0.309 | 6.67 | 33.11 | 1.73 | 8.57 |
| 0.5 | A | 0.230 | 0.180 | 2.81 | 28.55 | 0.89 | 9.08 |
| 0.5 | CBg | 0.009 | -0.001 | 0.15 | 23.35 | 0.11 | 15.92 |
| 0.5 | Cg | -0.003 | -0.002 | 0.05 | 36.05 | 0.05 | 32.40 |
| 0.5 | Cr | -0.002 | -0.002 | 0.07 | 23.98 | 0.06 | 15.90 |
| 5 | O | 0.817 | 0.681 | 10.33 | 29.24 | 2.84 | 8.03 |
| 5 | AE | 0.063 | 0.050 | 1.34 | 33.53 | 0.37 | 9.03 |
| 5 | Eg | 0.015 | 0.012 | 0.17 | 9.27 | 0.16 | 8.67 |
| 5 | Bg | 0.003 | -0.002 | 0.09 | 10.74 | 0.09 | 10.34 |
| 5 | Bs | -0.001 | -0.010 | 0.07 | 9.25 | 0.08 | 8.98 |
| 60 | O | 0.559 | 0.464 | 7.87 | 39.35 | 3.03 | 14.57 |
| 60 | AE | 0.255 | 0.160 | 2.12 | 25.04 | 1.26 | 14.92 |
| 60 | EAg | 0.010 | 0.029 | 0.47 | 15.98 | 0.34 | 11.57 |
| 60 | Bg | -0.004 | -0.001 | 0.06 | 5.93 | 0.06 | 6.84 |
| 60 | 2Bs | 0.003 | 0.003 | 0.09 | 4.89 | 0.10 | 5.54 |
| 60 | 2C | -0.001 | -0.020 | 0.04 | 17.83 | 0.05 | 20.97 |
| 120 | O | 0.235 | 0.202 | 16.13 | 73.19 | 5.92 | 26.86 |
| 120 | A | 0.402 | 0.330 | 2.21 | 33.96 | 1.58 | 24.30 |
| 120 | Er | 0.004 | -0.023 | 0.06 | 13.91 | 0.07 | 17.94 |
| 120 | Eg | -0.024 | -0.035 | 0.04 | 8.77 | 0.06 | 13.74 |
| 120 | Bg | -0.029 | -0.031 | 0.04 | 7.00 | 0.06 | 10.56 |

**Table S3:** Average atomic% concentrations (at.%) of elements at mineral surfaces in heavy fractions of selected topsoils (A and AE horizons) as analyzed by X-ray photoelectron spectroscopy. Abbreviation: BD = below detection limit. The percentage occupation of mineral surfaces by organic matter (OM) was deduced from the sum of carbon and oxygen atomic% concentrations; the OM-derived oxygen was estimated following the approach of Brodowski et al.^15^: O bound to carbon (OC) was differentiated from O bound to mineral-derived cations according to OC = O − (0.5Na + 1.5Al + 2Si + 0.5K + 1Ca + 1.176Fe).

|  | 0.5 ky |  |  | 5 ky |  |  | 60 ky |  |  | 120 ky | |  |
| --- | --- | --- | --- | --- | --- | --- | --- | --- | --- | --- | --- | --- |
|  |  |  |  |  |  |  |  |  |  |  | |  |
|  | at.% | Mean range |  | at.% | Mean range |  | at.% | Mean range |  | at.% | Mean range | |
|  |  |  |  |  |  |  |  |  |  |  |  | |
| C | 43.36 | 0.49 |  | 33.45 | 4.61 |  | 47.52 | 0.39 |  | 41.70 | 1.31 | |
| N | 1.66 | 0.03 |  | 0.96 | 0.13 |  | 0.94 | 0.08 |  | 1.08 | 0.08 | |
| O | 38.20 | 0.42 |  | 43.10 | 1.84 |  | 35.41 | 0.49 |  | 38.96 | 1.37 | |
| Si | 10.95 | 0.57 |  | 14.03 | 1.58 |  | 12.37 | 0.16 |  | 16.32 | 0.00 | |
| Al | 4.27 | 0.36 |  | 6.65 | 0.90 |  | 3.05 | 0.02 |  | 1.68 | 0.16 | |
| Ca | 0.12 | 0.17 |  | BD | ― |  | 0.12 | 0.01 |  | BD | ― | |
| K | 0.56 | 0.05 |  | 0.68 | 0.18 |  | BD | ― |  | BD | ― | |
| Na | 0.75 | 0.02 |  | 0.77 | 0.14 |  | 0.61 | 0.04 |  | 0.27 | 0.03 | |
| Fe | 0.15 | 0.00 |  | 0.25 | 0.06 |  | BD | ― |  | BD | ― | |
| **OM** | **53** |  |  | **38** |  |  | **53** |  |  | **45** |  | |

**Table S4:** Radiocarbon data of topsoil and subsoil heavy fractions from diagnostic horizons of selected sites.

| Site age (ky) | Horizon | pMC (%) | err (%) | ∆^14^C (‰) | err (‰) |
| --- | --- | --- | --- | --- | --- |
| 0.5 | A | 108.26 | 0.31 | 74.6 | 3.1 |
| 0.5 | CBg | 86.54 | 0.23 | -141.1 | 2.3 |
| 0.5 | Cg | 29.86 | 0.12 | -703.6 | 1.2 |
| 0.5 | Cr | 62.46 | 0.19 | -380.0 | 1.9 |
| 5 | AE | 100.02 | 0.44 | -7.2 | 4.4 |
| 5 | Eg | 83.42 | 0.22 | -172.1 | 2.2 |
| 5 | Bwg | 77.78 | 0.20 | -228.1 | 2.0 |
| 5 | Bos | 78.09 | 0.20 | -224.9 | 2.0 |
| 60 | AE | 106.74 | 0.27 | 59.4 | 2.7 |
| 60 | EAg | 95.40 | 0.23 | -53.1 | 2.3 |
| 60 | Bwg | 67.60 | 0.20 | -329.1 | 2.0 |
| 60 | Bos | 71.44 | 0.19 | -291.0 | 1.9 |
| 60 | 2C | 44.60 | 0.15 | -557.3 | 1.5 |
| 120 | A | 106.50 | 0.27 | 57.0 | 2.7 |
| 120 | Er | 53.03 | 0.18 | -473.7 | 1.8 |
| 120 | Eg | 20.42 | 0.12 | -797.3 | 1.2 |
| 120 | Bg | 11.21 | 0.10 | -888.8 | 1.0 |

| Aliphatic C | Methoxyl C | O-Alkyl C | Aryl C | Carbonyl and Amide C |
| --- | --- | --- | --- | --- |
| 0-46 ppm | 46-58 ppm | 58-110 ppm | 110-160 ppm | 160-190 ppm |
| 19 | 7 | 56 | 13 | 5 |
| 25 | 5 | 57 | 10 | 3 |
| 21 | 4 | 59 | 12 | 4 |
| 26 | 4 | 53 | 13 | 4 |
| 24 | 4 | 54 | 15 | 3 |
| 23 | 4 | 56 | 14 | 3 |
| 20 | 5 | 58 | 14 | 3 |

**Fig. S1**: ^13^C-NMR spectroscopy data of surface litter samples collected at the different sites. Values shown in the table depict the percentage contribution of major carbon species.





**Fig. S2:** Plot of (**a**) oxalate-extractable Fe plus Al (Fe_o_+Al_o_) and (**b**) pyrophosphate-extractable Fe and Al (Fe_p_+Al_p_) versus the percentage of OC in topsoil and subsoil bulk soils mineralized in 90 days at 25 °C under oxic and anoxic conditions. Data from oxic and anoxic incubations were each fitted by a simple exponential decay model (y=a×e^–bx^). Dashed lines indicate that raw data without transformation were not normally distributed. Errors bars depict standard deviation of replicates.

**Fig. S3:** Mineralization of light fraction material isolated from A horizons along the Franz Josef chronosequence by density fractionation and incubated over 125 days under oxic conditions at 15 °C. Details are given in Turner et al. (2017)^8^.

**

**

**Fig. S4:** (**a**) General relationship between the percent modern carbon (pMC) of heavy fractions and soil depth considering samples from the 0.5, 5, 12, and 120 ky site and (**b**) relationship of pMC values of topsoil heavy fractions and their contents of lignin-derived phenols (VSC), amino sugars (AS), and amino acids (AA).

**Kruskall-Wallis-ANOVA and post-hoc test results**

Site age groups 2-7 correspond to site ages of 0.5 to 120 ky. At the youngest site no A horizons existed as only transition OA horizons formed, which were classified as O horizons. Note, groups sizes only ranged between 2 and 4 (group 2: n = 2; group 3: n = 3; group 4: n = 3; group 5: n = 2;. group 6: n = 4; group 7: n = 4).

**
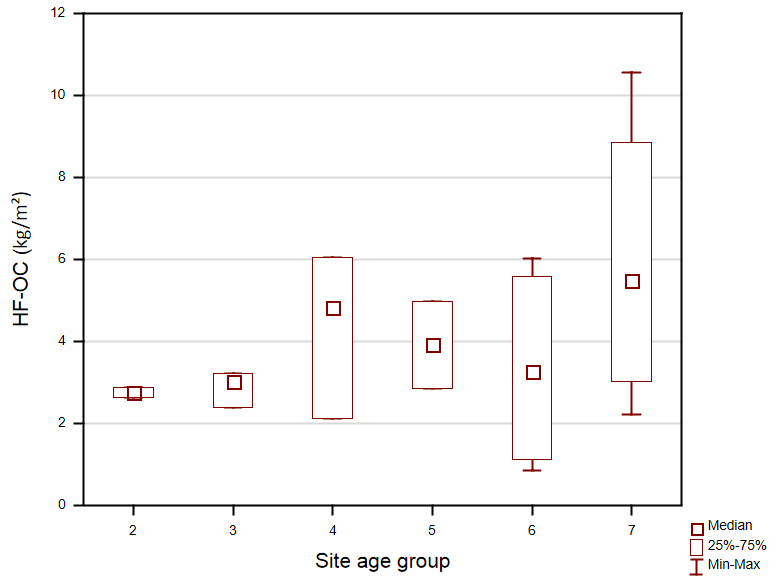
***
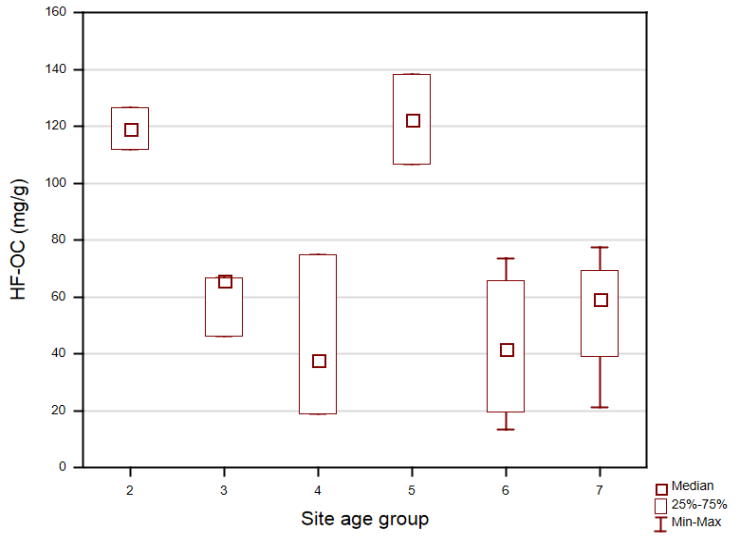
***ANOVA S1**: *Differences of mineral-associated organic carbon (OC) concentrations and stocks in topsoils and OC/ON ratios of topsoil mineral-associated organic matter. Abbreviation: HF, heavy fraction (mineral-associated)*

| Dep. Var.  HF-OC (mg/g) | Multiple comparisons p values (2-sided)  Indep. Var. Site age group  Kruskal-Wallis-test: H ( 5, N= 18) =9.450292 p =.0924 | | | | | |
| --- | --- | --- | --- | --- | --- | --- |
|  | \| 2 R:16.500 \| \| --- \| | \| 3 R:9.0000 \| \| --- \| | \| 4 R:6.6667 \| \| --- \| | \| 5 R:16.500 \| \| --- \| | \| 6 R:6.2500 \| \| --- \| | \| 7 R:8.2500 \| \| --- \| |
| \| 2 \| \| --- \| |  | 1.000000 | 0.654248 | 1.000000 | 0.399320 | 1.000000 |
| \| 3 \| \| --- \| | 1.000000 |  | 1.000000 | 1.000000 | 1.000000 | 1.000000 |
| \| 4 \| \| --- \| | 0.654248 | 1.000000 |  | 0.654248 | 1.000000 | 1.000000 |
| \| 5 \| \| --- \| | 1.000000 | 1.000000 | 0.654248 |  | 0.399320 | 1.000000 |
| \| 6 \| \| --- \| | 0.399320 | 1.000000 | 1.000000 | 0.399320 |  | 1.000000 |
| \| 7 \| \| --- \| | 1.000000 | 1.000000 | 1.000000 | 1.000000 | 1.000000 |  |

| Dep. Var.  HF-OC (kg m^-2^) | Multiple comparisons p values (2-sided)  Indep. Var. Site age group  Kruskall-Wallis-test: H (5; N = 18) = 2.345029, p = 0.7996 | | | | | |
| --- | --- | --- | --- | --- | --- | --- |
|  | \| 2 R:7.0000 \| \| --- \| | \| 3 R:8.0000 \| \| --- \| | \| 4 R:10.333 \| \| --- \| | \| 5 R:10.000 \| \| --- \| | \| 6 R:8.0000 \| \| --- \| | \| 7 R:12.500 \| \| --- \| |
| \| 2 \| \| --- \| |  | 1.000000 | 1.000000 | 1.000000 | 1.000000 | 1.000000 |
| \| 3 \| \| --- \| | 1.000000 |  | 1.000000 | 1.000000 | 1.000000 | 1.000000 |
| \| 4 \| \| --- \| | 1.000000 | 1.000000 |  | 1.000000 | 1.000000 | 1.000000 |
| \| 5 \| \| --- \| | 1.000000 | 1.000000 | 1.000000 |  | 1.000000 | 1.000000 |
| \| 6 \| \| --- \| | 1.000000 | 1.000000 | 1.000000 | 1.000000 |  | 1.000000 |
| \| 7 \| \| --- \| | 1.000000 | 1.000000 | 1.000000 | 1.000000 | 1.000000 |  |

**
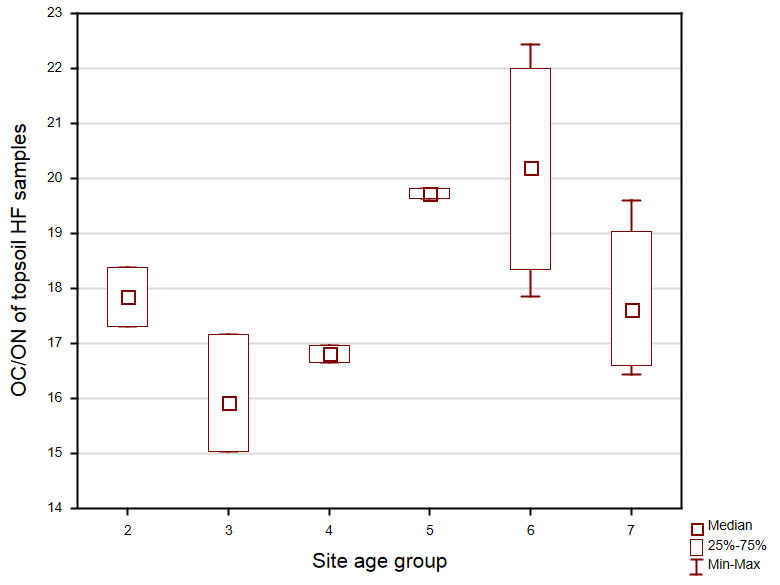
**

| Dep. Var.  HF-OC/ON | Multiple comparisons p values (2-sided)  Indep. Var. Site age group  Variable: Group Kruskal-Wallis-Test: H (5, N= 18) =11.32164 p =.0454 | | | | | |
| --- | --- | --- | --- | --- | --- | --- |
|  | \| 2 R:10.000 \| \| --- \| | \| 3 R:3.6667 \| \| --- \| | \| 4 R:5.6667 \| \| --- \| | \| 5 R:15.500 \| \| --- \| | \| 6 R:14.500 \| \| --- \| | \| 7 R:8.5000 \| \| --- \| |
| \| 2 \| \| --- \| |  | 1.000000 | 1.000000 | 1.000000 | 1.000000 | 1.000000 |
| \| 3 \| \| --- \| | 1.000000 |  | 1.000000 | 0.227642 | 0.118281 | 1.000000 |
| \| 4 \| \| --- \| | 1.000000 | 1.000000 |  | 0.654248 | 0.454180 | 1.000000 |
| \| 5 \| \| --- \| | 1.000000 | 0.227642 | 0.654248 |  | 1.000000 | 1.000000 |
| \| 6 \| \| --- \| | 1.000000 | 0.118281 | 0.454180 | 1.000000 |  | 1.000000 |
| \| 7 \| \| --- \| | 1.000000 | 1.000000 | 1.000000 | 1.000000 | 1.000000 |  |

**ANOVA S2**: *Differences of mineral-associated biomarker concentrations and stocks in topsoil horizons. Abbreviations: VSC, lignin; AS, amino sugars; AA, amino acids.*


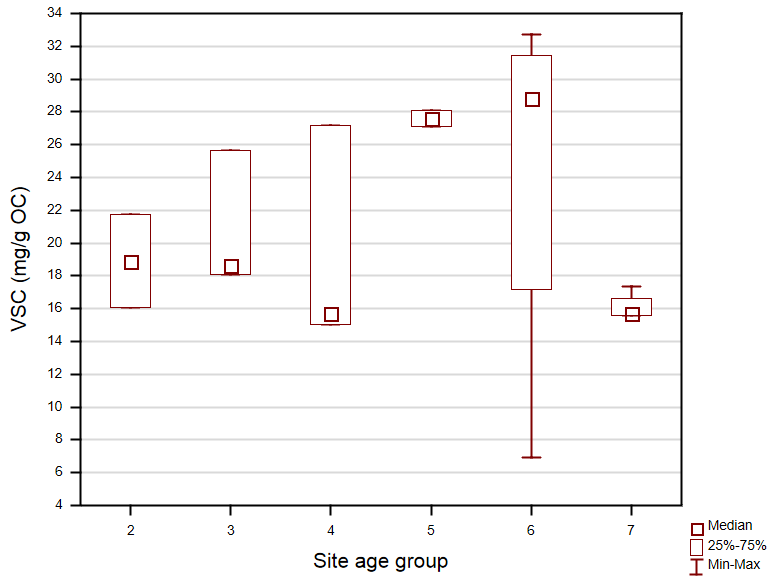

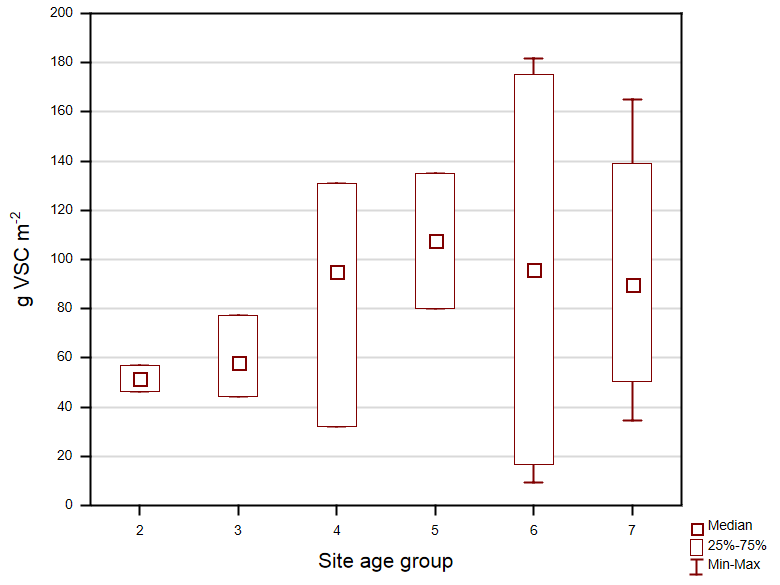


| Dep. Var.  VSC (mg g^-1^ OC) | Multiple comparisons p values (2-sided)  Indep. Var. Site age group  Kruskal-Wallis-Test: H (5, N= 18) =6.520468 p =.2588 | | | | | |
| --- | --- | --- | --- | --- | --- | --- |
|  | \| 2 R:9.0000 \| \| --- \| | \| 3 R:10.333 \| \| --- \| | \| 4 R:7.0000 \| \| --- \| | \| 5 R:14.500 \| \| --- \| | \| 6 R:12.750 \| \| --- \| | \| 7 R:5.2500 \| \| --- \| |
| \| 2 \| \| --- \| |  | 1.000000 | 1.000000 | 1.000000 | 1.000000 | 1.000000 |
| \| 3 \| \| --- \| | 1.000000 |  | 1.000000 | 1.000000 | 1.000000 | 1.000000 |
| \| 4 \| \| --- \| | 1.000000 | 1.000000 |  | 1.000000 | 1.000000 | 1.000000 |
| \| 5 \| \| --- \| | 1.000000 | 1.000000 | 1.000000 |  | 1.000000 | 0.681321 |
| \| 6 \| \| --- \| | 1.000000 | 1.000000 | 1.000000 | 1.000000 |  | 0.704171 |
| \| 7 \| \| --- \| | 1.000000 | 1.000000 | 1.000000 | 0.681321 | 0.704171 |  |

| Dep. Var.  g VSC m^-2^ | Multiple comparisons p values (2-sided)  Indep. Var. Site age group  Kruskal-Wallis-Test: H (5, N= 18) =1.988304 p =.8508 | | | | | |
| --- | --- | --- | --- | --- | --- | --- |
|  | \| 2 R:6.5000 \| \| --- \| | \| 3 R:7.6667 \| \| --- \| | \| 4 R:9.6667 \| \| --- \| | \| 5 R:13.000 \| \| --- \| | \| 6 R:9.5000 \| \| --- \| | \| 7 R:10.500 \| \| --- \| |
| \| 2 \| \| --- \| |  | 1.000000 | 1.000000 | 1.000000 | 1.000000 | 1.000000 |
| \| 3 \| \| --- \| | 1.000000 |  | 1.000000 | 1.000000 | 1.000000 | 1.000000 |
| \| 4 \| \| --- \| | 1.000000 | 1.000000 |  | 1.000000 | 1.000000 | 1.000000 |
| \| 5 \| \| --- \| | 1.000000 | 1.000000 | 1.000000 |  | 1.000000 | 1.000000 |
| \| 6 \| \| --- \| | 1.000000 | 1.000000 | 1.000000 | 1.000000 |  | 1.000000 |
| \| 7 \| \| --- \| | 1.000000 | 1.000000 | 1.000000 | 1.000000 | 1.000000 |  |


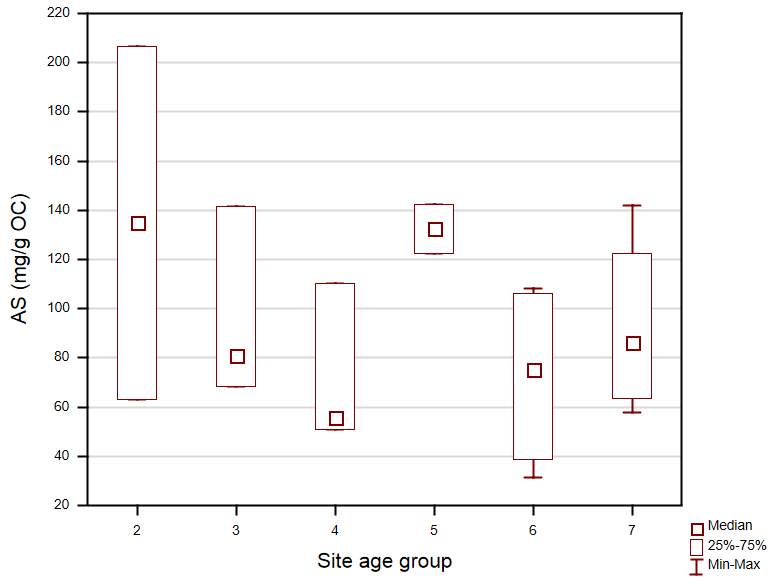

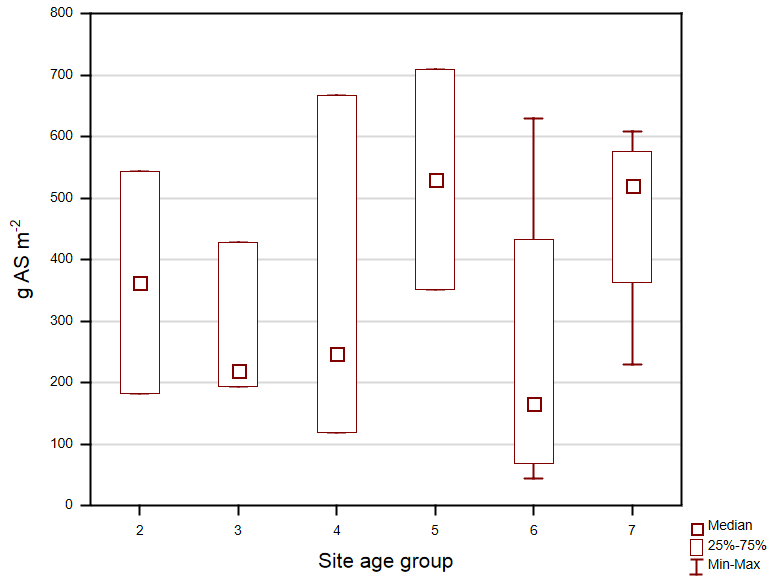


| Dep. Var.  AS (mg g^-1^ OC) | Multiple comparisons p values (2-sided)  Indep. Var. Site age group  Kruskal-Wallis-Test: H (5, N= 18) =5.154971 p =.3973 | | | | | |
| --- | --- | --- | --- | --- | --- | --- |
|  | \| 2 R:12.000 \| \| --- \| | \| 3 R:10.333 \| \| --- \| | \| 4 R:6.6667 \| \| --- \| | \| 5 R:15.500 \| \| --- \| | \| 6 R:6.5000 \| \| --- \| | \| 7 R:9.7500 \| \| --- \| |
| \| 2 \| \| --- \| |  | 1.000000 | 1.000000 | 1.000000 | 1.000000 | 1.000000 |
| \| 3 \| \| --- \| | 1.000000 |  | 1.000000 | 1.000000 | 1.000000 | 1.000000 |
| \| 4 \| \| --- \| | 1.000000 | 1.000000 |  | 1.000000 | 1.000000 | 1.000000 |
| \| 5 \| \| --- \| | 1.000000 | 1.000000 | 1.000000 |  | 0.773638 | 1.000000 |
| \| 6 \| \| --- \| | 1.000000 | 1.000000 | 1.000000 | 0.773638 |  | 1.000000 |
| \| 7 \| \| --- \| | 1.000000 | 1.000000 | 1.000000 | 1.000000 | 1.000000 |  |

| Dep. Var.  g AS m^-2^ | Multiple comparisons p values (2-sided)  Indep. Var. Site age group  Kruskal-Wallis-Test: H (5, N= 18) =3.926901 p =.5600 | | | | | |
| --- | --- | --- | --- | --- | --- | --- |
|  | \| 2 R:8.5000 \| \| --- \| | \| 3 R:7.3333 \| \| --- \| | \| 4 R:9.6667 \| \| --- \| | \| 5 R:14.000 \| \| --- \| | \| 6 R:6.7500 \| \| --- \| | \| 7 R:12.000 \| \| --- \| |
| \| 2 \| \| --- \| |  | 1.000000 | 1.000000 | 1.000000 | 1.000000 | 1.000000 |
| \| 3 \| \| --- \| | 1.000000 |  | 1.000000 | 1.000000 | 1.000000 | 1.000000 |
| \| 4 \| \| --- \| | 1.000000 | 1.000000 |  | 1.000000 | 1.000000 | 1.000000 |
| \| 5 \| \| --- \| | 1.000000 | 1.000000 | 1.000000 |  | 1.000000 | 1.000000 |
| \| 6 \| \| --- \| | 1.000000 | 1.000000 | 1.000000 | 1.000000 |  | 1.000000 |
| \| 7 \| \| --- \| | 1.000000 | 1.000000 | 1.000000 | 1.000000 | 1.000000 |  |


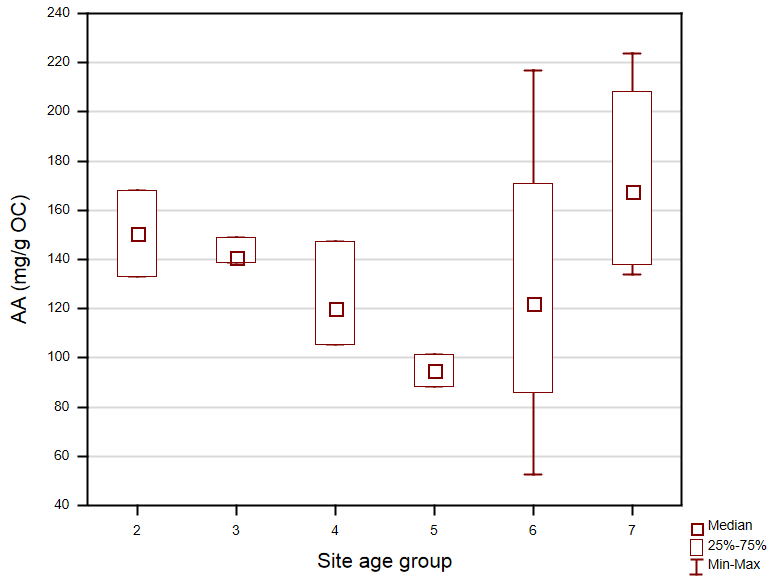

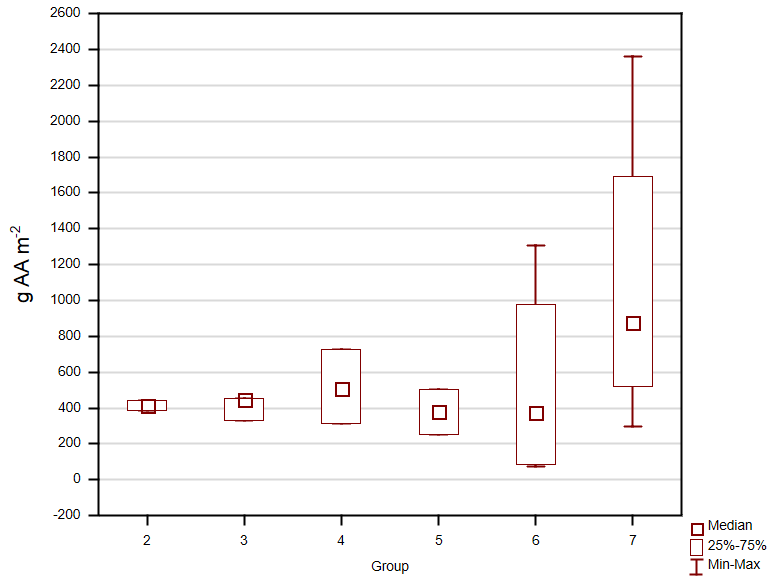


| Dep. Var.  AA (mg g^-1^ OC) | Multiple comparisons p values (2-sided)  Indep. Var. Site age group  Kruskal-Wallis-Test: H (5, N= 18) =7.663743 p =.1758 | | | | | |
| --- | --- | --- | --- | --- | --- | --- |
|  | \| 2 R:11.500 \| \| --- \| | \| 3 R:11.667 \| \| --- \| | \| 4 R:7.6667 \| \| --- \| | \| 5 R:2.5000 \| \| --- \| | \| 6 R:7.5000 \| \| --- \| | \| 7 R:13.750 \| \| --- \| |
| \| 2 \| \| --- \| |  | 1.000000 | 1.000000 | 1.000000 | 1.000000 | 1.000000 |
| \| 3 \| \| --- \| | 1.000000 |  | 1.000000 | 0.899660 | 1.000000 | 1.000000 |
| \| 4 \| \| --- \| | 1.000000 | 1.000000 |  | 1.000000 | 1.000000 | 1.000000 |
| \| 5 \| \| --- \| | 1.000000 | 0.899660 | 1.000000 |  | 1.000000 | 0.224415 |
| \| 6 \| \| --- \| | 1.000000 | 1.000000 | 1.000000 | 1.000000 |  | 1.000000 |
| \| 7 \| \| --- \| | 1.000000 | 1.000000 | 1.000000 | 0.224415 | 1.000000 |  |

| Dep. Var.  g AS m^-2^ | Multiple comparisons p values (2-sided)  Indep. Var. Site age group  Kruskal-Wallis-Test: H (5, N= 18) =3.128655 p =.6802 | | | | | |
| --- | --- | --- | --- | --- | --- | --- |
|  | \| 2 R:7.5000 \| \| --- \| | \| 3 R:8.3333 \| \| --- \| | \| 4 R:10.333 \| \| --- \| | \| 5 R:7.0000 \| \| --- \| | \| 6 R:8.2500 \| \| --- \| | \| 7 R:13.250 \| \| --- \| |
| \| 2 \| \| --- \| |  | 1.000000 | 1.000000 | 1.000000 | 1.000000 | 1.000000 |
| \| 3 \| \| --- \| | 1.000000 |  | 1.000000 | 1.000000 | 1.000000 | 1.000000 |
| \| 4 \| \| --- \| | 1.000000 | 1.000000 |  | 1.000000 | 1.000000 | 1.000000 |
| \| 5 \| \| --- \| | 1.000000 | 1.000000 | 1.000000 |  | 1.000000 | 1.000000 |
| \| 6 \| \| --- \| | 1.000000 | 1.000000 | 1.000000 | 1.000000 |  | 1.000000 |
| \| 7 \| \| --- \| | 1.000000 | 1.000000 | 1.000000 | 1.000000 | 1.000000 |  |

**Spearman rank correlation coefficients between the proportion of mineralizable carbon in bulk soils and selected soil variables.**

Bold values indicate significant relations at the 0.05 probability level. Incubations were run for 90 days at 25°C under oxic and anoxic conditions as described above. Sample numbers with *N* = 21 include O, A, E, B, and C horizons whereas O horizons were excluded when *N* = 17. Abbreviations: Fe_d_ = content of dithionite-extractable Fe (total pedogenic Fe), Fe_o_ = oxalate-extractable Fe (Fe in poorly crystalline Fe oxides and Fe-organic complexes; Fe_p_ = pyrophosphate-extractable Fe = Fe in Fe-organic complexes; Al_o_ = oxalate-extractable Al (Al in poorly crystalline Al phases and Al-organic complexes; Al_p_ = Al in Al-organic complexes); HF = heavy fraction (>1.6 g cm^–3^); LF = light fraction (<1.6 g cm^–3^); VSC = lignin-derived phenols; AS = hydrolysable amino sugars; AA = hydrolysable amino acids.

| Variable | N | Mineralized OC (%)  Anoxic | Mineralized OC (%)  Oxic |
| --- | --- | --- | --- |
| Mineralized C anox. (%) | 21 | 1.00 | **0.51** |
| Mineralized C ox. (%) | 21 | **0.51** | 1.00 |
| pH | 21 | 0.11 | -0.52 |
| Mean depth (cm) | 17 | 0.16 | -0.60 |
| Sand (%) | 17 | 0.16 | 0.32 |
| Silt (%) | 17 | -0.12 | -0.23 |
| Clay (%) | 17 | **-0.57** | **-0.49** |
| Fe_d_ (mg g^–1^) | 17 | **-0.77** | **-0.77** |
| Fe_o_+Al_o_ (mg g^–1^) | 17 | **-0.84** | **-0.80** |
| Al_o_ (mg g^–1^) | 17 | **-0.69** | **-0.84** |
| Fe_o_ (mg g^–1^) | 17 | **-0.85** | **-0.62** |
| Fe_p_+Al_p_ (mg g^–1^) | 17 | **-0.76** | **-0.62** |
| Al_p_ (mg g^–1^) | 17 | **-0.69** | **-0.76** |
| Fe_p_ (mg g^–1^) | 17 | **-0.71** | -0.33 |
| Δ^14^C (‰) | 17 | -0.20 | 0.43 |
| OC/ON bulk | 21 | -0.08 | 0.04 |
| OC/ON HF | 17 | **-0.73** | -0.44 |
| OC/ON LF | 17 | -0.09 | -0.71 |
| HF-OC (mg g^–1^) | 17 | -0.47 | 0.13 |
| VSC (mg g^–1^ OC) | 17 | -0.10 | **0.46** |
| AS (mg g^–1^ OC) | 17 | 0.22 | -0.02 |
| AA (mg g^–1^ OC) | 17 | -0.19 | **0.55** |
| δ^13^C HF (‰) | 17 | 0.42 | -0.27 |
| δ^13^C LF (‰) | 17 | 0.21 | **-0.58** |
| δ^15^N HF (‰) | 17 | -0.29 | **-0.85** |
| δ^15^N LF (‰) | 17 | -0.10 | 0.10 |

**References**

1. Wardle, D. A., Walker, L. R. & Bardgett, R. D. Ecosystem properties and forest decline in contrasting long-term chronosequences. *Science* **305**, 509–513 (2004).

2. Walker, T. W. & Syers, J. K. The fate of phosphorus during pedogenesis. *Geoderma* **15**, 1–19 (1976).

3. Menge, D. N. L. & Hedin, L. O. Nitrogen fixation in different biogeochemical niches along a 120 000-year chronosequence in New Zealand. *Ecology* **90**, 2190–2201 (2009).

4. Dietel, J. *et al.* Complexity of clay mineral formation during 120,000 years of soil development along the Franz Josef chronosequence, New Zealand. *N. Z. J. Geol. Geophys.* **60**, 23–35 (2017).

5. Richardson, S. J., Peltzer, D. A., Allen, R. B., McGlone, M. S. & Parfitt, R. L. Rapid development of phosphorus limitation in temperate rainforest along the Franz Josef soil chronosequence. *Oecologia* **139**, 267–276 (2004).

6. European Space Agency. *Sentinel-2 User Handbook*. (2015).

7. Turner, S. *et al.* Mineralogical impact on long-term patterns of soil nitrogen and phosphorus enzyme activities. *Soil Biol. Biochem.* **68**, 31–43 (2014).

8. Turner, S. *et al.* Microbial community dynamics in soil depth profiles over 120,000 years of ecosystem development. *Front. Microbiol.* **8**, (2017).

9. Golchin, A., Oades, J., Skjemstad, J. & Clarke, P. Study of free and occluded particulate organic matter in soils by solid state ^13^C CP/MAS NMR spectroscopy and scanning electron microscopy. *Soil Res* **32**, 285–309 (1994).

10. Mikutta, R. *et al.* Biogeochemistry of mineral–organic associations across a long-term mineralogical soil gradient (0.3–4100 kyr), Hawaiian Islands. *Geochim. Cosmochim. Acta* **73**, 2034–2060 (2009).

11. Zhang, X. & Amelung, W. Gas chromatographic determination of muramic acid, glucosamine, mannosamine, and galactosamine in soils. *Soil Biol. Biochem.* **28**, 1201–1206 (1996).

12. Brodowski, S., Amelung, W., Lobe, I. & Preez, C. C. D. Losses and biogeochemical cycling of soil organic nitrogen with prolonged arable cropping in the South African Highveld — evidence from D- and L-amino acids. *Biogeochemistry* **71**, 17–42 (2005).

13. Loftfield, N., Flessa, H., Augustin, J. & Beese, F. Automated gas chromatographic system for rapid analysis of the atmospheric trace gases methane, carbon dioxide, and nitrous oxide. *J. Environ. Qual.* **26**, 560–564 (1997).

14. Mikutta, R. *et al.* Biodegradation of forest floor organic matter bound to minerals via different binding mechanisms. *Geochim. Cosmochim. Acta* **71**, 2569–2590 (2007).

15. Brodowski, S., Amelung, W., Haumaier, L., Abetz, C. & Zech, W. Morphological and chemical properties of black carbon in physical soil fractions as revealed by scanning electron microscopy and energy-dispersive X-ray spectroscopy. *Geoderma* **128**, 116–129 (2005).
